# Mismatch between perceived family and individual chronotype and their association with sleep-wake patterns

**DOI:** 10.1038/s41598-019-43168-9

**Published:** 2019-05-01

**Authors:** Angela J. Pereira-Morales, Ana Adan, Leandro P. Casiraghi, Andrés Camargo

**Affiliations:** 1 Program in Public Health, School of Medicine, Universidad Nacional de Colombia, Bogotá, Colombia; 2 Department of Clinical Psychology and Psychobiology, School of Psychology, University of Barcelona, Barcelona, Spain; 3 Institute of Neurosciences, University of Barcelona, Barcelona, Spain; 4 Department of Biology, University of Washington, Seattle, WA 98195 USA; 5 School of Medicine, Universidad de Ciencias Aplicadas y Ambientales. U.D.C.A, Bogotá, Colombia

**Keywords:** Population screening, Epidemiology

## Abstract

While social *zeitgebers* are known to shape diurnal preference, little research has been devoted to determining the contribution of the familiar group chronotype as social zeitgeber on individual circadian rhythms and sleep-wake patterns in adult subjects. The current study aimed to examine the matching between perceived family chronotype and individual chronotype and their relationship with sleep-wake patterns on weekdays and weekends, diurnal subjective somnolence, and substance consumption. Nine hundred and forty-two Colombian adults completed the Composite Scale of Morningness, the Epworth Sleepiness Scale, and responded to a questionnaire about circadian preferences of their family nucleus. We found evidence of a mismatch between perceived family and individual chronotype, mainly for morning-type individuals (Cohen’s Kappa = −0.231; p < 0.001). This mismatch was associated with diurnal subjective somnolence (β = 0.073; p < 0.001) and specific sleep-wake patterns (p < 0.01). In addition, subjects with evening-type families showed higher caffeine and alcohol consumption (p < 0.001). To our knowledge, this is the first study to assess and report the mismatching between perceived family and individual chronotypes, and it adds to the existing body of knowledge regarding the influence of social *zeitgebers* on circadian rhythms. This is particularly relevant since mismatching between circadian physiology and environmental cues have been shown to lead to diverse pathologies.

## Introduction

Circadian rhythms play a key role in human physiology by precisely regulating sleep and wake periods, appetite, cognitive function, and mood states, among many other functions^1^. While the temporal patterns of circadian functions follow a stable phase synchrony with daily cues (known as *zeitgebers*) and between each other, interindividual differences in said phase relationships have been described in humans. Based on their preferred timing of active and rest periods, people can be classified generally into three diurnal preference types or “chronotypes”: morning-type, neither-type, or evening-type^2,3^.

A person’s chronotype depends on several factors: genetic^4^, environmental^5^, as well as constitutional (i.e. age and sex) variables^6^. Older age correlates with a tendency to morningness, while young adults and teenagers are more often inclined to eveningness^7^. Sex differences are mainly linked to endocrine factors^8^, with males showing an evening preference more often than women^9^.

Environmental cues, most importantly the light-dark cycle, as well as social demands like school and working schedules are relevant for the determination of diurnal preference and its possible consequences in terms of health and social adaptations^10^. Familial interactions present a very specific type of social cueing. However, little research has been conducted in order to determine a potential contribution of this factor to individual chronotype.

Familiar effects can be mediated by genetic and heritable traits. Genetic studies have reported associations between extreme morningness with the Familial Advanced Sleep Phase Syndrome (ASPS) and of extreme eveningness with the Delayed Sleep Phase Syndrome (DSPS), though the results have been discordant^11^. In addition, a rural Brazilian family-based cohort study using the Morningness-Eveningness Questionnaire (MEQ), reported evidence of heritability of the MEQ score of 38% controlling for age, gender, and geographical zone of residence^12^. Overall, studies suggest that heritability accounts for between 12.4 and 29.4% of the variance in chronotype^13^, while 50% is determined by genetic factors^14^.

However, there can also be an effect of family derived from social behavior and individual chronotypes interactions per se. Some studies have looked at women during and after pregnancy and analyzed the interactions between the newborn’s, mother’s and father’s sleep patterns and chronotypes and they found that mother-infant matching is rooted in biological pacemakers and it predicts children’s social rhythms, and that new parent’s social rhythms showed changes on 24‐hr sleep‐wake rhythm after birth^15,16^.

Other studies have focused on the relationship between diurnal preferences of parents and their children. Boergers *et al*. reported an association between children’s sleep problems and parental chronotype, while Leonhard *et al*. found that children act as a social factor affecting mothers’ chronotype^17,18^. However, to our knowledge, no studies have addressed the relationship between family circadian preferences and individual chronotypes in adult subjects.

Additionally, substances consumption has been found relevant for sleep-wake patterns in samples of adults and young adults. Specifically, caffeine and alcohol consumption has been related to shorter sleep duration, increased sleep onset latency, and increased daytime sleepiness^19,20^. Smoking also has been associated with an increased long sleep latency, sleep medication use, and daytime sleepiness^21^.

The aim of the present study was to assess the possible matching or mismatching between perceived family and individual chronotype and their relationship with sleep and wake patterns, and sleep problems in a large sample of Colombian adults, including substances consumption as independent factors.

## Methods

### Participants

This study was approved by the Institutional Ethics Committee of the Universidad de Ciencias Aplicadas y Ambientales (Bogotá, Colombia) which certified its compliance with the ethical principles for medical research involving human subjects. Nine hundred and forty-two Colombian subjects participated in the study (mean age: 21.8; ±SD 5.0; range: 18–49; 63.7% females) after signing an informed written consent form. No monetary or academic incentive was given to the subjects for participation in the study. All participants resided in Bogotá, Colombia (04 °38′N, 74°05′W), and were recruited from two private universities in the same city. The sample was composed of two main groups: students (n = 786; mean age: 20.8; ±SD 2.9) and institutional workers (n = 156; mean age: 27.9; ±SD 8.6). Subjects’ inclusion criteria involved the absence of a personal history of neurological disorders, and of any sleep disorder (or current sleep pharmacological treatment) or chronic somatic diseases. The sample of this study was composed in part by subjects also included in two previous studies^22,23^.

### Measures

#### Chronotype

Individual chronotype was assessed through the Composite Scale of Morningness (CSM), which is a 13-items Likert-type scale widely used to assess behavioral temporal preference and to classify individuals in the dimension of morningness-eveningness^24^. The CSM has been shown to be highly valid and reliable across cultures^25^. We used the translated version to the Spanish language performed previously by Adan *et al*.^26^, which has shown be valid and consistent in Colombian samples^23,27^. Based on previous studies performed on local populations, the following criteria were used to determine chronotypes based on the CSM score: morning-type: 36 points or below; evening-type: 43 points or over; subjects scoring between 37 and 42 points were considered as neither-type^22^. The CSM can be dissected into three main factors^3^. The *Morningness* factor is composed by CSM items 1, 3, 6, 8, 10, and 11 (scores range from 6 to 25), with higher scores reflecting the effort of getting up early in the morning. The *Morning alertness* factor is composed by items 4, 5, and 12 (scores range from 3 to 12), with higher scores reflecting a high level of alertness during the first half hour after waking up. The *Activity plan* factor is composed by the items 2, 7, 9, and 13 (scores range from 4 to 18), with higher scores indicating a self-perception of a high level of morning activity and an early bedtime. This three-factor solution was corroborated in the current sample through an exploratory factorial analysis and was found a Cronbach’s alpha of 0.71 for the whole scale.

To assess the perceived “family chronotype” participants were asked the following question: *How do you think the people that make up your family nucleus (e.g. parents, brothers, sisters, children, grandparents) are, regarding their sleep habits?”* Participants were required to choose within three possible answers: *“early risers”* (morning-types), *“night owls”* (evening-types), or *“indifferent”* (neither-types). The participants were asked to consider their “family nucleus” as the people in his family with whom they currently lived.

#### Sleep-wake patterns

To assess the sleep-wake pattern of the participants, we asked them to inform the number of hours of nocturnal sleep (HNS) per day during weekdays and weekends, with the following question: *“How many hours do you sleep per night on weekdays and weekends?”*

#### Subjective diurnal somnolence

General subjective level of diurnal somnolence was measured with the Epworth Sleepiness Scale (ESS), an auto-administered questionnaire that evaluates dozing behavior in different daily life situations^28^. Its score ranges from 0 to 24 (from no to high somnolence). The validity of the ESS has been previously reported for a Colombian population^29^. The current study revealed a Cronbach’s alpha of 0.71 for the instrument.

#### Substances consumption

Participants were asked to report whether they regularly consumed 1) caffeinated drinks, 2) tobacco, and 3) alcoholic drinks. Only *yes* or *no* were recorded as answers.

### Statistical analysis

After checking the normality of data through skewness and kurtosis, we examined the variance differences in HNS in weekdays and weekends and of diurnal subjective somnolence included as dependent variables, according to sex, occupation, individual chronotype and perceived family chronotype as independent/fixed factor variables.

In order to test statistical differences in the sociodemographic variables, HNS in weekdays and weekends, diurnal subjective somnolence, and scores in the CSM subscales were treated as dependent variables while perceived family chronotype was treated as the independent variable. Analysis of variance was performed for continuous outcome variables considering the partial Eta squared (*η*^2^_*p*_) as effect size. Tukey’s honestly significant difference (HSD) post hoc test was used and a p-value of 0.016 was set for the post-hoc tests. For the categorical variables (sex, caffeine, and alcohol consumption and smoking), chi-squared tests were used.

Cohen’s Kappa coefficient was utilized to examine inter-rater reliability and accuracy of agreement between the individual and perceived family chronotypes. To test mean differences for concordance between the perceived family and individual chronotypes and sleep variables, we carried out an independent samples *t*-test and calculated effect sizes through Cohen’s *d*. In addition, to examine the association between sleep variables separately for the morning and evening participants whose chronotype did not match that of their families, Pearson’s correlation analyses were conducted. Because the sample was predominantly composed of young adults and the most frequently expected case was that of evening-type subjects along morning-type families, the above analyses were reduced to these two chronotypes.

Finally, in order to explore the association between individual and familiar chronotypes a categorical regression analysis (CATREG) version 3.0 (DTSS, Leiden, The Netherlands) was used, controlling for relevant sociodemographic variables. CATREG analysis is appropriate for models in which there are categorical variables given that each categorical variable introduced in the model is transformed by means of replacing categories with optimal values using the optimal scaling methodology. CATREG estimates the regression coefficients and the quantifications simultaneously in an iterative process; furthermore, this analysis uses Lasso (Least Absolute Shrinkage and Selection Operator), a regularized regression method that has demonstrated be more accurately for the prediction of the dependent variable through a subset of more stable predictors. Regularized regression comprises shrinking of the regression coefficients to improve the prediction accuracy by means of the reduction of standard errors of the coefficients estimates in the presence of multicollinearity^30^. Two CATREG models were conducted, the first for morning and evening types and the second for the “non-matching” group of participants. In both models, the outcome variable of the analysis was the individual chronotype, included as a categorical variable in the model, while the predictor variables were perceived family chronotype, sex, age, caffeine and alcohol consumption, and smoking included as categorical variables, and subjective diurnal somnolence as a continuous variable. For the individual and perceived family chronotypes only the morning- and evening-types were analyzed, for the reasons discussed above.

All statistical analyses were performed using the Statistical Package for the Social Sciences V.22 (SPSS Inc., Chicago, IL). The statistical significance was set at *p* values < 0.05. Data are presented as *mean* ± *standard deviation*, when applicable.

## Results

Average of reported hours of nocturnal sleep per day during weekdays was 5.5 h ± 1.5 and 8.6 h ± 2.2 on weekends. The average of diurnal subjective somnolence for the whole sample was 10.1 ± 4.2. Descriptive data for the reported HNS per day during weekdays, as well as for diurnal subjective somnolence is shown in Table 1. No statistical differences were found according to sex or individual chronotype for HNS per day. Significant differences were found in HNS during weekdays according to occupation, with workers reporting a higher average of hours of nocturnal sleep (F _(1, 939)_ = 8.48; p = 0.003; *η*^2^_*p*_ = 0.009). Perceived family chronotype was found to significantly affect reported hours of sleep during weekdays, with neither-types displaying the highest average of HNS (F _(2, 938)_ = 5.93; p = 0.002; *η*^2^_*p*_ = 0.013). Regarding diurnal subjective somnolence, women showed a higher average levels than males (F _(1, 940)_ = 30.8; p = 0.0000; *η*^2^_*p*_ = 0.032), as also did students compared with workers (F _(1, 940)_ = 6.41; p = 0.011; *η*^2^_*p*_ = 0.007), and evening-types among chronotypes (F _(2, 939)_ = 10.41; p = 0.0001; *η*^2^_*p*_ = 0.022). In addition, subjects reporting a morning-type family showed a higher average of diurnal subjective somnolence (F _(2, 938)_ = 3.40; p = 0.033; *η*^2^_*p*_ = 0.007) compared with subjects reporting other perceived family chronotypes.

**Table 1:** Descriptive data and statistical contrasts for hours of nocturnal sleep and diurnal subjective somnolence according to sex, occupation, individual chronotype, and family chronotype.

	Weekdays (SD)	Hours of nocturnal sleep			Diurnal subjective somnolence	
		F (df)	Weekends (SD)	F (df)	Mean (SD)	F (df)
Females Males	5.53 (1.60) 5.49 (1.32)	0.11 (1, 940)	8.58 (2.16) 8.71 (2.19)	0.81 (1, 940)	10.76 (4.31) 9.17 (4.03)	30.8** (1, 940)
Work (yes/not)	5.83/5.45 (1.34/1.53)	8.48** (1, 940)	8.66/8.61 (2.06/2.19)	0.07(1, 940)	10.04/10.22 (4.18/4.29)	0.23 (1, 940)
Study (yes/not)	5.46/6.17 (1.52/1.16)	14.36** (1, 940)	8.60/8.91 (2.19/1.87)	1.32 (1, 940)	10.28/8.93 (4.29/3.99)	6.41* (1, 940)
**Individual chronotype**						
Morning-type	5.67 (1.34)	2.03 (2, 932)	8.46 (2.12)	3.00 (2, 932)	9.35 (4.35)	10.41** (2, 932)
Evening-type	5.43 (1.54)		8.89 (2.33)		11.01 (4.07)	
Neither-type	5.47 (1.58)		8.56 (2.07)		10.18 (4.26)	
**Perceived family chronotype**						
Morning-type	5.20 (1.35)	5.93** (2, 932)	8.61 (2.05)	1.18 (2, 932)	10.89 (4.13)	3.40* (2, 932)
Evening-type	5.58 (1.53)		8.56 (2.20)		9.98 (4.33)	
Neither-type	5.68 (1.54)		8.85 (2.18)		10.03 (4.19)	

Note: *p < 0.05; **p < 0.01; df = degrees of freedom.

The overall average CSM score for the whole sample was 39.63 ± 5.51. Subjects reported their chronotype as follows: 270 morning-types (28.7%; 165 females, 105 males), 269 evening-types (28.6%; 181 females, 88 males), and 403 neither-types (42.8%; 254 females, 149 males). Perceived family chronotypes as reported by participants revealed 194 to be morning-types (20.6%), 577 evening-types (61.3%), and 170 neither types (18.1%) (Table 2).

**Table 2:** Sociodemographic characteristics according to family and individual chronotype.

	Perceived family chronotype			Statistics
	Morning-type (n = 191)	Neither-type (n = 98)	Evening-type (n = 577)	
Age (years)	21.61 ± 4.64	22.22 ± 5.79	21.83 ± 4.87	F_(2,940)_ = 0.61
Sex (females) n (%)	135 (14.3)	98 (10.4)	366 (38.9)	*x*^2^ = 5.61
Caffeine consumption (yes) n (%)	153 (16.3)	116 (12.3)	399 (42.4)	*x*^2^ = 7.41*
Alcohol consumption (yes) n (%)	98 (10.4)	53 (5.6)	231 (24.6)	*x*^2^ = 13.9**
Smoking (yes) n (%)	44 (4.7)	27 (2.9)	91 (9.7)	*x*^2^ = 5.12
HNS in weekdays	5.2 ± 1.3	5.6 ± 1.5	5.5 ± 1.5	F_(2,932)_ = 5.93**
HNS in weekends Family chronotype*HNS in weekdays and weekends	8.6 ± 2.0	8.8 ± 2.2	8.6 ± 2.2	F = 1.18 F_(2,932)_ = 3.92**
Diurnal subjective somnolence (ESS)	10.89 ± 4.13	10.03 ± 4.19	9.98 ± 4.33	F_(2,932)_ = 3.40*
**CSM Factors**				
Morningness	17.59 ± 3.16	18.60 ± 2.85	18.71 ± 3.02	F_(2,932)_ = 10.22**
Activity planning	13.19 ± 2.26	12.72 ± 2.38	11.51 ± 2.64	F_(2,932)_ = 35.99**
Morning Alertness	8.42 ± 1.87	8.52 ± 1.98	8.06 ± 1.90	F_(2,932)_ = 3.35*
**Individual chronotype**				
	**Morning-type (n** = **269)**	**Neither-type (n** = **401)**	**Evening-type (n** = **265)**	**Statistics**
Age (years)	23.61 ± 7.10	21.35 ± 3.78	20.86 ± 3.35	F_(2,940)_ = 25.12**
Sex (females) n (%)	165 (17.5)	254 (27.0)	181 (10.2)	*x*^2^ = 2.35
Caffeine consumption (yes) n (%)	178 (18.9)	294 (31.2)	197 (20.9)	*x*^2^ = 4.77
Alcohol consumption (yes) n (%)	80 (8.5)	168 (17.9)	134 (14.2)	*x*^2^ = 23.4**
Smoking (yes) n (%)	39 (4.1)	65 (6.9)	58 (6.2)	*x*^2^ = 5.35
HNS in weekdays	5.67 ± 1.34	5.47 ± 1.58	5.43 ± 1.54	F_(2,932)_ = 2.03
HNS in weekends Chronotype*HNS in weekdays and weekends	8.46 ± 2.12	8.56 ± 2.07	8.89 ± 2.33	F_(2,932)_ = 3.00 F_(2,932)_ = 3.98**
Diurnal subjective somnolence (ESS)	9.35 ± 4.35	10.18 ± 4.26	11.01 ± 4.07	F_(2,932)_ = 10.41**
**CSM Factors**				
Morningness	21.46 ± 1.95	18.55 ± 1.81	15.31 ± 2.23	F_(2,932)_ = 647.3**
Activity planning	14.61 ± 1.80	12.76 ± 1.93	10.81 ± 2.22	F_(2,932)_ = 245.7**
Morning Alertness	9.85 ± 1.55	8.31 ± 1.52	6.88 ± 1.51	F_(2,932)_ = 254.8**

Note: **p < 0.01; *p < 0.05; % are reported according to the total sample. ESS: Epworth somnolence scale. CSM: Composite scale of morningness. HNS: Hours of nocturnal sleep.

According to variance analysis, we found significant differences between reported perceived family chronotype in HNS in weekdays and weekends, diurnal subjective somnolence, and in all CSM subscales (*Morningness*, *Activity planning*, and *Morning alertness*; Table 2). Tukey’s HSD post hoc test showed that morning-types reported less HNS than evening-types and neither types (Mean difference: MD = 0.38; p < 0.01; MD = 0.49; p < 0.01; respectively). For the *Morningness* factor of the CSM, participants with morning-type families revealed lower scores when compared with evening-type families (MD = 1.12; p < 0.01) and with neither-type families (MD = 1.01; p < 0.01). Those with evening-type families and with neither type families showed higher scores for the *Activity planning factor* when compared with those with morning-type families (MD = 1.66; p < 0.01; MD = 1.22; p < 0.01; respectively). Regarding the *Morning alertness factor* a small albeit overall significant difference was found according to familiar chronotype (F = 3.35; p = 0.035; *η*^2^_*p*_ = 0.007), however, a post-hoc test did not show significant differences between groups.

Table 1 shows the variance and frequency analyses for sociodemographic variables and CSM factors according to individual chronotype. Statistical differences were found for individual chronotype according to alcohol consumption, HNS in weekdays and weekends, diurnal subjective somnolence, and each of the three CSM factors. Specifically, the post-hoc analyses of HNS in weekdays and weekends did not show statistical differences. Diurnal subjective somnolence presented a difference between morning- and evening-types (MD = −1.67; p < 0.01). Regarding CSM factors, all contrasts were significant (p < 0.01). Neither- and evening-types reported a higher alcohol consumption than morning-types (p < 0.01).

The overall Cohen’s kappa, the measure of inter-rater reliability and agreement for individual and perceived family chronotype, was −0.231 (Table 3). Based on published guidelines^31^ this kappa value suggests only minimal agreement, although statistically significant (p < 0.001). Morning- and evening-type participants whose chronotype matched that of their families (as perceived by themselves) represented only 38.3% of the sample, while “non-matching” subjects represented the 61.6%. In the “non-matching” participants group individual chronotypes were 34.3% morning-types and 10.7% evening-types; while perceived family chronotypes were 23.4% morning-types and 62.8% evening-types.

**Table 3:** Matching/not matching between perceived family and individual chronotype.

Perceived family chronotype	Morning-type n%		Evening-type n%		*Statistic* Cohen’s Kappa coefficient
Individual chronotype					
Morning-type	31	7.0	191	43.1	This divisory line it is not necessary, because cohen’s kappa is for both morning-type and evening -type
Evening-type	82	18.5	139	31.4	−0.231**

**p < 0.001.

The “non-matching” participants displayed significantly lower levels of diurnal subjective somnolence compared with “matching” participants, although with a small effect size (Cohen’s d = 0.10). No differences were observed for the average HNS during weekdays nor during weekends (Table 4). Furthermore, “non-matching” subjects displayed higher mean values at all CSM subscales, and these differences showed medium effect sizes (Table 4).

**Table 4:** Mean differences and effect sizes for the sleep-wake patterns according to concordance between perceived family and individual chronotype for morning and evening types subjects.

	Match (n = 170)		No match (n = 273)		t	Effect size Cohen’s d
	M	SD	M	SD		
Diurnal subjective somnolence	10.85	4.24	9.88	4.43	2.27*	0.10
HNS during weekdays	5.45	1.58	5.60	1.35	1.09	0.05
HNS during weekends	8.79	2.39	8.56	2.17	1.03	0.04
***CSM Factors***						
Morningness	16.26	3.05	19.58	3.64	9.90**	0.42
Activity planning	11.85	2.26	13.25	3.01	5.22**	0.24
Morning alertness	7.46	1.96	8.86	2.03	7.14**	0.32

Note: *p < 0.05; **p < 0.01; M = mean; SD: standard deviation. CSM: Composite scale of morningness. HNS: Hours of nocturnal sleep.

The Pearson’s correlation analysis for the non-matching participants showed significant negative associations between diurnal subjective somnolence with the CSM *morningness factor* (p < 0.01) and with the *morning alertness factor* (p < 0.01). Also, we found significant positive associations between HNS during weekdays and all CSM factors (p < 0.05) (Supplementary material, Table S1).

A CATREG analysis revealed that the model including perceived family chronotype can explain 11% of the variance in individual chronotype (morning- and evening-types) (Table 5). Perceived family chronotype explains on its own 6.8% of the variance (β = 0.26; p < 0.01). Age, alcohol consumption, diurnal subjective sleepiness and hours of nocturnal sleep during weekends also correlated significantly with individual chronotype in the whole sample. In the CATREG analysis only including the “non-matching” subjects, the perceived family chronotype can explain 72.5% of the variance in individual chronotype (morning- and evening-types). Perceived family chronotype explains on its own 71% of the variance (β = 0.84; p < 0.01). Age and diurnal subjective somnolence were significant variables in the model (Table 5).

**Table 5:** Categorical regressions for perceived family chronotype, sociodemographic information, and subjective diurnal sleepiness, taking individual chronotype as the categorical outcome variable.

Variable	Β	SE	R^2^
***Model 1: Sample of morning and evening chronotypes***			0.14
Perceived family chronotype	0.197**	0.046	
Sex	0.068	0.041	
Age	−0.154**	0.048	
Caffeine consumption	0.056	0.039	
Alcohol consumption	0.143**	0.048	
Smoking	0.077	0.044	
Diurnal subjective somnolence	0.105*	0.047	
Working	0.019	0.036	
HNS during weekdays	−0.053	0.050	
HNS during weekends	0.126*	0.050	
***Model 2: Not matching sample (Morning and evening types)***			0.72
Perceived family chronotype	0.806**	0.021	
Sex	0.008	0.02	
Age	−0.100**	0.033	
Caffeine consumption	0.002	0.017	
Alcohol consumption	0.04	0.027	
Smoking	0.016	0.021	
Diurnal subjective somnolence	0.073**	0.026	
Working	0.009	0.021	
HNS during weekdays	0.049	0.028	
HNS during weekends	−0.008	0.032	

Note: p < 0.01; *p < 0.05; SE: standard error. Sex was coded as 1 = Female, 2 = Male; Chronotype was coded as 1 = morning-type, 2 = evening-type. Alcohol, smoking, and caffeine intake were coded as 1 = YES, 2 = NO.

## Discussion

To our knowledge, this is the first study to present evidence of a correlation between individual chronotype and reported familiar chronotype. The results of our work indicate that familiar interactions should be added to the list of the multiple factors that determine diurnal preference phenotype. We observed several differences in sleep patterns according to perceived family chronotype: individuals that considered their families as morning-type reported fewer hours of nocturnal sleep during weekdays and a higher level of subjective sleepiness during the day. In addition, perceived family chronotype was found to be an important explicative factor on individual chronotype variance.

All three CSM subscales showed significant association with reported familiar chronotype. We found that individuals whose chronotypes did not match with their perceived family chronotype (excluding neither-types) showed higher values in the CSM subscales, suggesting that these individuals could have a tendency to be “early risers”. This finding is concordant with the fact that in the “non-matching” participants group the predominant individual chronotype was the morning type (34.3%), while the predominant perceived family chronotype was the evening type (62.8%). At this point, it is interesting to highlight previous results from a Colombian study, showing significant associations between PER2 gene polymorphisms with higher values of CSM-morning alertness factor and between PER3 gene polymorphisms with higher values of CSM-morningness factor^22^. Future studies could address this genetic variables in relation to the self-reports used in this study, considering that these genes have a central role in the molecular mechanism of the circadian rhythms.

Our findings are in line with those reported by Salgado *et al*. regarding sleep disturbances associated with disruption of circadian rhythms by lifestyle conditions^32^. In the first place, we found that diurnal sleepiness scores were higher for participants who considered their families to be “morning-types”, and this was the group that presented the lesser level of agreement between individual and familiar chronotype (only 7%). Individuals who matched their own with their families’ chronotype reported higher average levels of somnolence, although this seemingly paradoxical result may arise as an indirect effect of these being mainly evening-type individuals, which showed greater subjective somnolence values. On the other hand, when we conducted a correlation analysis only for the group of the “non-matching” subjects (including only evening- and morning-types individuals), significant negative associations were found between diurnal subjective somnolence and the CSM *Morningness* and *Morning Alertness* factors. This finding shows the impact of having an evening preference on diurnal alertness when the perceived family chronotype does not match with the individual chronotype. In addition, the association found between HNS in weekdays and all CSM factors strengthens this interpretation, showing an apparent attempt of the non-matching individuals to compensate the sleep debt and diurnal somnolence of the week with more hours of sleep on weekends.

Furthermore, previous studies also found a significant relationship between diurnal subjective somnolence evening preference chronotype^33,34^. If we assume both the subject and her family to prefer to go to bed late while demands of study and work requirements that they wake up early we can hypothesize that sleep timing will not be satisfying or even appropriate, leading to increased sleepiness during the day. In this sense, we found that a high proportion of evening-type subjects and of subjects reporting evening-type families sleep fewer hours than other chronotypes during weekdays, and sleep more hours on weekends, which may suggest that evening-types try to compensate on the weekends the few hours of sleep achieved during the weekdays. In addition, this need for sleep compensation could be more important when both the subject and her family are evening-types.

We found that participants with perceived evening-type families reported more often to consume caffeinated and alcoholic beverages. This result fits in with reports indicating a higher prevalence of substance use by evening-type subjects, being considered this chronotype as a risk factor in the onset and maintenance of drug consumption^35–37^. Moreover, a relevant hypothesis to consider is that having an evening chronotype added to family mismatch could represent an increased risk factor. However, it is important to mention that a relevant limitation of the current study was the assessment of substance consumption which was in a dichotomous form (*yes* or *no*), therefore the consumption could not be quantified.

Mismatch of natural circadian rhythmicity and individual chronotype with natural and/or social zeitgebers has been proven deleterious to human health at several levels including mental health^38,39^. Our work has addressed the subject of “familiar” diurnal preference understood as an additional social *zeitgeber* affecting human daily rhythms, beyond those widely studied as work and school schedules and social-cultural interactions. As such, it could be considered as an element in therapies aiming to stabilize social rhythms, according to the “social *zeitgeber”* theory^40^.

Some limitations of the present study should be addressed. More importantly, this work consisted of a cross-sectional design relaying in undetailed self-reports of the several variables studied and family-chronotype was assessed through the subjects’ own perception. Moreover, the effect sizes of our results were relatively small. In this sense, the inclusion of a standardized assessment of social rhythm disruption or the individual chronotype of family members would have increased the weight of our findings. Moreover, while the absence of a diagnosis of the mental disease was included as an inclusion criterion, we did not record any depressive and anxiety symptomatology measures. However, the size of our sample is large enough to support the general outline of our findings. Also, importantly, our data add to previous literature in the field, focused mainly in European and North American samples, by including a previously non-studied population from South America.

Our findings suggest that mismatching between perceived family and individual chronotypes represents a relevant factor affecting biological rhythmicity that should be addressed in future studies. More detailed studies dissecting each of the variables in this work through precise measurements should be conducted in order to determine the exact contributions of familiar chronotype on individual circadian health and disease.

## Supplementary information

Pearson’s correlations for the sub-group of non-matching participants

## Data Availability

The datasets generated and analyzed during the current study are available from the corresponding author on reasonable request.
