# Supplementary material for: Mismatch between perceived family and individual chronotype and their association with sleep-wake patterns
Source: Sci Rep. 2019 May 1;9:6756. doi: 10.1038/s41598-019-43168-9 (PMC6494859; doi:10.1038/s41598-019-43168-9)
Supplement: Supplementary file 1 — Pearson’s correlations for the sub-group of non-matching participants [file 41598_2019_43168_MOESM1_ESM.docx]

**Mismatch between perceived family and individual chronotype and their association with sleep-wake patterns**

Angela J. Pereira-Morales, BSc (1); Ana Adan, Ph.D. (2,3); Leandro P. Casiraghi, Ph.D (4); Andrés Camargo*, RN, MSc, (5)

*Corresponding author

1. PhD Program in Public Health, School of Medicine, Universidad Nacional de Colombia, Bogotá, Colombia.

2. Department of Clinical Psychology and Psychobiology, School of Psychology, University of Barcelona, Barcelona, Spain.

3. Institute of Neurosciences, University of Barcelona, Barcelona, Spain.

4. Department of Biology, University of Washington, Seattle, WA 98195, USA.

5. School of Medicine, Universidad de Ciencias Aplicadas y Ambientales. U.D.C.A, Bogotá, Colombia. Email: [andcamargo@udca.edu.co](mailto:andcamargo@udca.edu.co).

**Table S1.** Pearson´s correlations for the sub-group of non-matching participants

|  | HNS in weekends | ESS | Morningness | Activity planning | Morning Alertness |
| --- | --- | --- | --- | --- | --- |
| HNS in weekdays | 0.385** | -0.070 | 0.134* | 0.275** | 0.145* |
| HNS in weekends |  | 0.023 | -0.066 | 0.010 | 0.011 |
| Diurnal subjective somnolence (ESS) |  |  | -0.230** | -0.101 | -0.242** |

Note: **p<0.01; *p<0.05
